# Antifungal Combinations in Dermatophytes

**DOI:** 10.3390/jof7090727

**Published:** 2021-09-05

**Authors:** Lucia Brescini, Simona Fioriti, Gianluca Morroni, Francesco Barchiesi

**Affiliations:** 1 Dipartimento di Scienze Biomediche e Sanità Pubblica, Università Politecnica delle Marche, 60020 Ancona, Italy; l.brescini@univpm.it (L.B.); s.fioriti@staff.univpm.it (S.F.); g.morroni@univpm.it (G.M.); 2 Malattie Infettive, Azienda Ospedaliera Ospedali Riuniti Marche Nord, 61121 Pesaro, Italy

**Keywords:** dermatophytes, antifungals, antifungal susceptibility testing, drug combinations

## Abstract

Dermatophytes are the most common cause of fungal infections worldwide, affecting millions of people annually. The emergence of resistance among dermatophytes along with the availability of antifungal susceptibility procedures suitable for testing antifungal agents against this group of fungi make the combinatorial approach particularly interesting to be investigated. Therefore, we reviewed the scientific literature concerning the antifungal combinations against dermatophytes. A literature search on the subject performed in PubMed yielded 68 publications: 37 articles referring to in vitro studies and 31 articles referring to case reports or clinical studies. In vitro studies involved over 400 clinical isolates of dermatophytes (69% *Trichophyton* spp., 29% *Microsporum* spp., and 2% *Epidermophyton floccosum*). Combinations included two antifungal agents or an antifungal agent plus another chemical compound including plant extracts or essential oils, calcineurin inhibitors, peptides, disinfectant agents, and others. In general, drug combinations yielded variable results spanning from synergism to indifference. Antagonism was rarely seen. In over 700 patients with documented dermatophyte infections, an antifungal combination approach could be evaluated. The most frequent combination included a systemic antifungal agent administered orally (i.e., terbinafine, griseofulvin, or azole—mainly itraconazole) plus a topical medication (i.e., azole, terbinafine, ciclopirox, amorolfine) for several weeks. Clinical results indicate that association of antifungal agents is effective, and it might be useful to accelerate the clinical and microbiological healing of a superficial infection. Antifungal combinations in dermatophytes have gained considerable scientific interest over the years and, in consideration of the interesting results available so far, it is desirable to continue the research in this field.

## 1. Introduction

Dermatophytes are the most common cause of fungal infections worldwide, affecting millions of people annually. Dermatophytes are filamentous fungi with the ability to invade keratinised tissue, such as skin, hair, and nails [1]. Classically, they are divided into three genera: *Trichophyton, Epidermophyton,* and *Microsporum* [2]. However, this classification is based on the phenotype of the species and led to misclassification of morphological mutants. In 2017, de Hoog et al. constructed a phylogenetic tree using sequences of the nuclear ribosomal internal transcribed spacers (ITS rDNA) and divided the dermatophytes into seven clades: *Trichophyton, Epidermophyton, Nannizzia, Paraphyton, Lophophyton, Microsporum*, and *Arthroderma* [3]. Based on their host specificity, these fungi are classified into three ecological groups: geophilic, zoophilic, and anthropophilic. Geophilic dermatophytes rarely cause infection in animals and humans but may be carried by animals in their fur. Zoophilic dermatophytes occur in the fur of animal hosts, either symptomatically or asymptomatically, and can be easily transmitted to humans. When zoophilic and geophilic species are transmitted to humans, they cause acute, inflammatory mycoses. Transmission of anthropophilic dermatophytes is usually from human to human. They cause chronic, mild, noninflammatory infections [4,5]. Ringworm or tinea is one of the most frequent clinical aspect of dermatophytosis. Among the tinea infections, tinea corporis, tinea cruris, tinea pedis, and onychomycosis are the most predominant types. The dermatophytes *T. rubrum*, *T. interdigitale* and *T. mentagrophytes*, are the main aetiological agents of dermatophytosis of skin and nails in humans [1,2,3,4,5].

Medical treatment of dermatophytosis consists of topical or oral antifungal agents. There are many topical agents for treating several less severe forms of tinea [6]. The azole derivatives, such as clotrimazole, miconazole, econazole, and oxiconazole, are the generally used. Agents from the allylamine family, such as terbinafine and naftifine, are also used. Other topical agents, such as ciclopirox or amorolfine, can be effective in the less severe cases of onychomycosis. In the more severe forms of dermatophyte infections, oral treatment is generally employed [6]. The first oral agent used to treat a dermatophyte infection was griseofulvin, introduced in clinical practice in 1958 [7]. This molecule interferes with microtubule formation, thus impairing fungal growth and cell division. Allylamines (mainly terbinafine) and triazoles (mainly itraconazole) are used for oral therapy. Both allylamines and triazoles act on the same cellular target, that is, the cell membrane. Triazoles inhibit sterol 14-α-demethylase, and allylamines inhibit squalene epoxidase, the inhibition of both enzymes leading to inhibition of ergosterol biosynthesis. Allylamines also lead to the accumulation of lanosterol, a toxic intermediary compound of the ergosterol biosynthesis pathway [8,9,10]. Terbinafine, which acts as a fungicide, is the drug of choice against *Trichophyton* spp. because of its clinical efficacy [11]. However, in the last years, an increasing incidence of chronic and recalcitrant dermatophytic infections have been described. Although rare, resistance to terbinafine has been documented among isolates of *T. rubrum* and *T. mentagrophytes/T. interdigitale* complex [12]. The resistance is generally due to several point mutations in the squalene target gene. This phenomenon, first described in recalcitrant dermatophytosis observed in India, was later reported in other countries [12,13,14,15,16,17]. Due to a very limited number of antifungals effective against dermatophytes and the emergence of resistance to these drugs, an in vitro antifungal susceptibility testing should be implemented in reference laboratories to monitor this phenomenon.

Currently, two standardized techniques for in vitro antifungal susceptibility testing of dermatophytes based on a broth microdilution procedure are available: one from the Clinical Laboratory Standards Institute (CLSI) and the other from the European Committee on Antimicrobial Susceptibility Testing (EUCAST) [18,19]. Although very similar, the two methods differ in endpoint determination. Lately, the EUCAST method was validated in a multicentre study (10 laboratories) in which terbinafine, itraconazole, voriconazole, and amorolfine were tested against a blinded panel of 38 terbinafine wild types and target gene mutant isolates of *T. rubrum* and *T. interdigitale.* The higher interlaboratory reproducibility was obtained using a medium with the addition of chloramphenicol and cycloheximide and measuring the MIC spectrophotometrically at 50% inhibition [20].

An antifungal combination strategy has been lately implemented to overcome the resistance phenomenon against a wide variety of infections due to either yeasts or filamentous fungi [21,22]. Achievement of a synergistic interaction is desirable in these contexts. The emergence of resistance among dermatophytes along with the availability of antifungal susceptibility procedures suitable for testing antifungal agents against this group of fungi make the combinatorial approach particularly interesting to be investigated. Therefore, we aimed to review the scientific literature concerning the antifungal combinations used against dermatophytes. In order to include most of the published papers on this topic, the revision was carried out using the classic dermatophyte nomenclature, which divides these fungi into three genera. In particular, the results of in vitro combinations of several antifungals or antifungals plus other chemical compounds are presented. Additionally, the effects of combinatorial regimens in human infections are reported.

## 2. Materials and Methods

**Figure 1:**
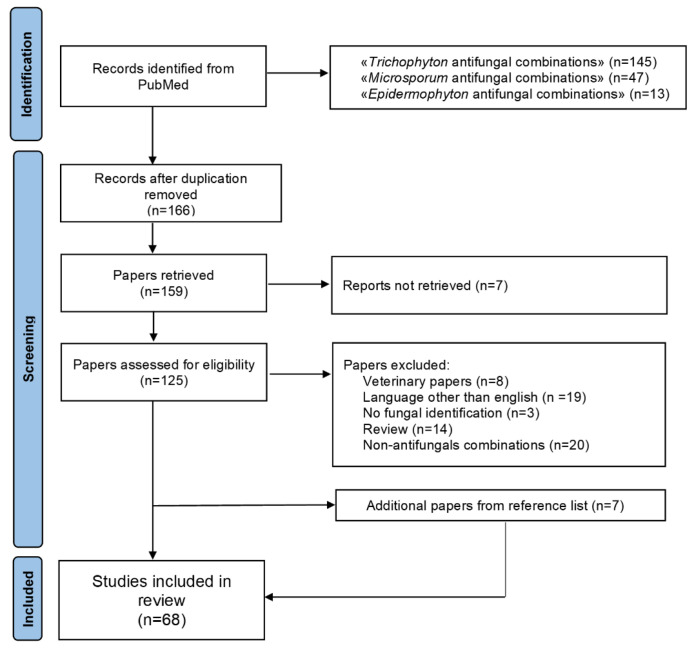
Flowchart of the different phases of article selection of the review.

This systematic review was conducted in accordance with the PRISMA guidelines [23] (Figure 1). PubMed was searched for dermatophytes antifungal combinations therapy with the following search string: “trichophyton” and “antifungal” and “combination”; “microsporum” and “antifungal” and “combination”; “epidermophyton” and “antifungal” and “combination”. Literature search was conducted on 1 June 2021, by three individual researchers (L.B., S.F., and G.M.). In case of discrepancies in the process of inclusion of papers/data extraction, a consensus was reached through discussion or involvement of a fourth reviewer (F.B.). Additional cases were sought from the reference list of included papers. The inclusion criteria were antifungal combinations for *Trichophyton* spp., *Microsporum* spp., and *Epidermophyton floccosum.* The exclusion criteria were papers not referring to human studies (i.e., veterinary cases), papers in languages other than English, unreachable publications, papers not specifying the genera/species of dermatophytes, reviews of the literature, combinations considering two chemical compounds other than antifungals, and combinations not considering chemical compounds (i.e., photodynamic therapy). Data from the included papers were entered in a database, created with Excel, which encompassed the genus/species/number of dermatophytes tested, the type of drug combination, the method utilized for testing, and the results of the interaction. In case of clinical reports, demographic data (when available) and outcome of the combination therapy were also reported.

## 3. Results and Discussion

A total of 205 articles were initially identified (Figure 1). After duplication removal, 166 articles were screened. Further exclusion included papers that were out of topic (34), veterinary (8), not in English (19), without fungal identification (3), literature reviews (14), and about combinations not including at least one antifungal agent (20). Additional 7 papers found in the reference list of the screened articles were added to the 61 eligible papers. Therefore, a total of 68 publications were included in this review: 37 articles referring to in vitro studies, and 31 articles referring to case reports or clinical studies (Tables 1–3). Among the first group of articles, there were 7 reports in which the combination of two antifungal agents was used, while 30 articles in which an antifungal agent was combined with a chemical compound other than an antifungal agent.

### 3.1. Antifungal Combinations

The results of in vitro antifungal combinations are reported in Table 1. *Trichophyton* spp. represented the most common genus tested, followed by *Microsporum* spp. and *E. floccosum.* Combinations included amorolfine plus azoles or terbinafine or griseofulvin; azoles plus griseofulvin or terbinafine; azoles plus ciclopirox [24,25,26,27,28,29,30]. Checkerboard titration methodology was the most common procedure for testing a combination (6/7 studies). Two studies investigating the effects of the combination of amorolfine or ciclopirox plus azoles found 100% synergistic interaction against many *Trichophyton* spp. [26,28]. One study confirmed this positive effect by adding two additional methods (disk-diffusion and E-test assays) to the broth microdilution procedure [26]. Although antagonism was never observed in any report, the type of interaction varies according to drug and isolate tested. In general, amorolfine plus azoles yielded synergistic interaction more often than amorolfine plus griseofulvin or plus terbinafine. One study investigated three new topical drugs (efinaconazole, tavaborole, and luliconazole) with itraconazole or terbinafine against *T. rubrum* and *T. interdigitale*. Efinaconazole with terbinafine or itraconazole exerted a synergistic effect on 43.8% and 12.5% of the strains tested, respectively. Conversely, luliconazole showed no synergistic effect with terbinafine but was synergistically effective with itraconazole against 31.3% of the strains. Tavaborole (an inhibitor of protein synthesis in fungal cells) showed no synergistic effect with terbinafine and was synergistically effective with itraconazole against 18.8% of the strains [29].

Overall, these data would suggest that an antifungal combination regimen might be useful against an infection due to dermatophytes. It is interesting to note that even combining drugs acting against a common fungal target (i.e., ergosterol—azoles, allylamines, and morpholine drugs such as amorolfine), a positive interaction in terms of reduction of the MIC of both drugs is often observed.

### 3.2. Antifungals Combined with Several Chemical Compounds

The results of in vitro activities of antifungals combined with other compounds are reported in Table 2. Again, *Trichophyton* spp. represented the most common genus tested. Combination included: azoles or terbinafine or griseofulvin plus plant extracts including essential oils (19/30 studies), azoles or terbinafine or amorolfine plus immunosuppressant agents (3 studies), azoles or terbinafine plus peptides (3 studies), azoles plus disinfectants (3 studies), and other combinations including antifungal agents plus efflux pump inhibitors and statins. Checkerboard titration methodology was the most common procedure for testing the combination, followed by agar methods (i.e., disk diffusion and agar dilution) [31,32,33,34,35,36,37,38,39,40,41,42,43,44,45,46,47,48,49,50,51,52,53,54,55,56,57,58,59,60].

It has been shown that plants have the capacity to produce secondary metabolites, including those which are constituents of essential oils, as defence mechanisms against herbivores and microorganisms. They act in two ways: by neutralizing free radicals (the antioxidant effect) and as anti-inflammatory agents by inhibiting the release of pro-inflammatory mediators. Secondary metabolites produced by plants are also capable of acting in a third way, as antifungal agents [31,32,33,34,35,36,37,38,39,40,41,42,43,44,45,46,47,48,49]. A synergistic interaction between antifungal agents and natural products was often seen (Table 2). One recent study evaluated the antifungal activity of tea tree oil (TTO) (*Melaleuca alternifolia* essential oil) and its main components against *T. rubrum* alone and in association with ketoconazole or itraconazole and showed either their fungicidal effects or a synergism upon combination with azoles [49]. Most of the studies demonstrated that the type of interaction was either isolate- or drug-dependent. One research assessed the antifungal activity of essential oil from *Mentha x piperita* against a wide panel of dermatophyte clinical isolates and found a fungistatic activity against these fungi. When this compound was used in combination with azoles, a synergic interaction was observed for *T. mentagrophytes* while indifference was detected for *T. rubrum* and *M. canis* [48]. Overall, these data would suggest that these natural compounds are one of the most promising sources for pharmacological research and that the development of new natural antimicrobial agents against many microbial pathogens, including dermatophytes, is warranted.

Calcineurin inhibitors (i.e., tacrolimus and cyclosporin A) or inhibitors of the mTOR pathway (i.e., sirolimus) are anti-rejection drugs widely used in organ transplant recipients and to prevent graft-versus-host disease in allogeneic stem cell recipients. However, these compounds also possess intrinsic antifungal activity against selected fungi [50,51,52]. One study evaluated the in vitro interactions between tacrolimus or triamcinolone acetate with itraconazole, terbinafine, bifonazole, and amorolfine against 28 clinical dermatophyte isolates, including 13 *T. rubrum*, 6 *T. mentagrophytes*, 5 *M. canis*, and 4 *E. floccosum* and found that a synergistic interaction was more often observed when the antifungal agents were combined with tacrolimus rather than cortisone [52]. Another study evaluated the combination of fluconazole with either tacrolimus or cyclosporine in an ex vivo *T. mentagrophytes* human skin infection model. Conidia colonization was monitored by scanning electron microscopy over a 7-day treatment period. The fluconazole–tacrolimus combination was superior to one single-drug therapy by clearing conidia and protecting skin from damage at low drug concentrations [50]. Similarly, when tacrolimus was added to itraconazole against 5 isolates of *T. mentagrophytes*, a synergistic interaction was observed in 80% of the cases [51]. Overall, these data indicate that calcineurin inhibitors are synergistic with ergosterol biosynthesis inhibitors against dermatophytes, and that a potential clinical application may be desirable.

Another interesting therapeutic approach might be represented by peptides because they act efficiently and rapidly against a wide range of pathogens including bacteria, fungi, viruses, and protozoa. Moreover, peptide resistant mutants rarely emerge with these molecules, especially when they are used in combination with other anti-infective drugs [53,54,55]. Among these compounds, protegrins and defensin were originally isolated from mammalian leucocytes. One study evaluated the in vitro effects of IB-367 alone and in combination with three antifungal drugs against 20 clinical isolates of dermatophytes belonging to three species and showed synergism in 35%, 30%, and 25% of IB-367/fluconazole, IB-367/itraconazole, and IB-367/terbinafine interactions, respectively. IB-367 exerted a fungicidal activity against *T. mentagrophytes*, *T. rubrum*, and *M. canis* at concentrations starting from 1 × MIC. At a concentration of 5 × MIC, IB-367 showed the highest rates of hyphae damage for *M. canis* and *T. mentagrophytes* [55]. Another study investigated the in vitro effects of tachyplesin III (TP), a potent disulphide-linked peptide, in combination with terbinafine against 20 clinical isolates of dermatophytes belonging to four species. Terbinafine in combination with TP showed indifferent activity against 14 of the 20 isolates (70%); synergic activity against 6 of them (30%); no antagonistic activity was observed [54]. Finally, the lipopeptide Pal-Lys-Lys-NH_2_ (PAL) alone and in combination with standard antifungal agents was tested against 24 clinical isolates of dermatophytes belonging to four species. Synergy was observed in 67%, 52%, and 15% of PAL/itraconazole, PAL/terbinafine, and PAL/fluconazole interactions, respectively. None of these combinations yielded antagonistic interactions. When synergy was not achieved, there was still a decrease in the MIC of one or both drugs used in the combination [53]. Overall, these studies demonstrated that peptides have potential activity against dermatophytes. These drugs, applied in the form of lacquer, spray, or ointment, could represent an interesting new therapy, particularly when combined with conventional treatment in recalcitrant or resistant dermatophyte infections.

Another combinatorial approach investigated the activity of an antifungal, generally miconazole, with the antiseptic compound chlorhexidine [56,57,58]. One study demonstrated that this association yielded a synergistic effect in vitro against 5 out of 10 isolates of *M. canis*, and an additive effect against 4 isolates, while when the same combination was tested against 9 isolates, each of *T. mentagrophytes* and *T. erinacei*, the most frequent interactions observed were additivism or indifference. Again, antagonism was never observed [57,58].

In general, the results obtained by combination of antifungal agents with chemical compounds other than antifungals yielded variable results spanning from synergism to indifference. Antagonism was rarely seen. This interaction is well documented for natural products (i.e., essential oils) as shown by a substantial number of scientific publications. Although promising results were documented, the different methods used to test these combinations hampered a univocal and comprehensive conclusion on the real effectiveness of these combinations.

### 3.3. Clinical Cases

The results of antifungal combinations in humans are reported in Table 3. There were 25 papers describing 37 single case reports, one paper each describing 36 and 254 patients, respectively, and 3 clinical trials involving a total of 410 patients [61,62,63,64,65,66,67,68,69,70,71,72,73,74,75,76,77,78,79,80,81,82,83,84,85,86,87,88,89,90,91]. Either paediatric or adult patients were represented. Tinea corporis, tinea capitis, and tinea unguium were the most common clinical manifestations. In the single case reports, the most frequent combination approach included a systemic antifungal agent administered orally (i.e., terbinafine, griseofulvin, or azole—mainly itraconazole) plus a topical medication (i.e., azole, terbinafine, ciclopirox, cortisone) for several weeks. Few cases were treated with both drugs given topically or orally. Only in two clinical cases, resistance mechanisms were assessed and confirmed by sequencing of the SQLE gene [67,70]. One patient with *Trichophyton* endophthalmitis and five patients with fungal keratitis due to *T. shoenleinii* were treated with a combination of systemic antifungal agents, including voriconazole or fluconazole, plus an antifungal agent administered topically (amphotericin B or miconazole). The outcome consisted in full recovery or improvement in most of the cases [74,86]. The 36 patients included in one paper consisted of 18 children and 18 adults with infections due to *T. violaceum*. The source of contagion was traced to 13 children, 11 African and 2 Ukrainian, adopted from an orphanage, with misdiagnosed tinea capitis. All 13 index cases and the 16 patients infected by them were treated with griseofulvin for 45 days and topical imidazoles. The adults with spreading tinea corporis were treated with 100 mg itraconazole for 15–20 days and those with tinea capitis with the same dose of the antimycotic for 45 days and with topical imidazoles. In all patients, recovery was confirmed by clinical and mycological examination 3 months after healing [87]. One early observational study involving 254 patients with various forms of dermatophyte infections mainly due to *Trichophyton* spp., concluded that topical treatment (Wilkinson’s salve, iodized alcohol 5%, undecylenic acid derivatives, 5-bromo-4′-chlorosalicylanilides, tolnaftates) plus griseofulvin possibly enhances the healing capacity and shortens the time for treatment, but it has no effect in preventing reinfections [88]. One randomized study of toenail onychomycosis with matrix area involvement due to *T. rubrum* in most cases, compared amorolfine 5% nail lacquer once weekly for 24 weeks given with 200 mg of itraconazole once daily for 6 or 12 weeks vs. itraconazole alone given for 12 weeks [91]. Combination therapy showed to be significantly more effective than monotherapy, both in terms of mycological and clinical cures at week 12. Similarly, another randomized study comparing amorolfine plus terbinafine vs. terbinafine alone in 249 patients with onychomycosis showed a significantly higher success rate for patients undergoing combination therapy relative to those in monotherapy at 18 months [89]. Another randomized study investigated the efficacy of combination therapy with oral griseofulvin and oral prednisolone to oral griseofulvin alone in the treatment of kerion celsi due to *Trichophyton* spp. [91]. Both groups were treated with oral griseofulvin for 8 weeks, whereas oral prednisolone was given in tapering doses for 3–4 weeks to the first group only. The final evaluation at week 12 showed a cure rate of 100% in both groups without any significant difference in terms of clinical or mycological cure.

## 4. Conclusions

Although dermatophyte infections are rarely life threatening, their chronicity and the frequency of relapse require prolonged treatment, resulting in an increased risk of drug toxicity and development of drug resistance. Similarly to what has been already observed in systemic fungal infections sustained by *Candida* spp. or *Aspergillus* spp., emergence of drug resistant strains among isolates of *Trichophyton* spp. has been lately documented.

Although dermatophytes are a group of fungi quite difficult to test in vitro (i.e., slow growth, inoculum preparation, incubation intervals etc.), standardized procedures have been introduced and validated, thereby making antifungal susceptibility testing of dermatophytes easier. This has led to experimenting with various pharmacological associations aimed at increasing the efficacy of the therapy against this group of fungi. Most of in vitro studies investigated the combination of classic antifungal agents with several, disparate, chemical compounds. The association between an antifungal drug and plant extracts, including essential oils, seems to evoke a particular interest. The reciprocal potentiation of the molecules upon combination makes these approaches particularly appealing in clinical practice. Although the intrinsic mechanisms of antifungal activity of these natural products have not been fully investigated, several cell targets are simultaneously involved, thereby making the occurrence of resistance unlikely. Clinical data indicate that an association of antifungal agents (systemic plus topic) is effective, and it might be useful in speeding up the clinical and microbiological healing of a superficial infection. It must be noted, however, that there are few controlled/randomized clinical trials and that unequivocal conclusions cannot be drawn. Another limitation is the lack of well-characterized antifungal-resistant isolates, whose treatment could especially benefit from a combination approach.

In summary, antifungal combinations against dermatophytes have gained considerable scientific interest over the years. To establish whether this approach can become a reliable treatment option, additional in vitro and clinical data are warranted.

## Figures and Tables

**Table 1:** Antifungal combinations against dermatophytes: in vitro effects of antifungal plus antifungal.

Reference	Number of Isolates and Species	Combinations	Methods	Reading Endpoint	Results
Banic et al., 1989 [24]	28 *M. canis*	GRI + KTZ	Growth in Broth; 28 °C, 168 h	% of inhibition	Some strains of *M. canis* were completely inhibited by GRI + KTZ
Harman et al., 2009 [25]	4 *T. rubrum*, 2 *T. mentag. var. interdigitale*, 2 *T. mentag. var. granulare*, *1 T. tonsurans*	AMF + TER/FLU/ITZ	Ck; 27 °C, 168 h	≥80% inhibition	Additivism or indifference
Laurent et al., 2017 [26]	9 *T. rubrum*	AMF + ITZ/KTZ/MIZ/SER/SUL	Ck, disk diffusion and E-test assay; 30 °C, 168 h	≥80% inhibition	Synergy: 100%
Polak et al., 1993 [27]	3 *T. mentagrophytes*, 1 *T. rubrum*, 2 *M. canis*,	AMF + ITZ/FLU/GRI/TER/KET	Agar dilution Ck; 30 °C, 96 h	No visible growth	Synergy: AMF + GRI 16%; AMF + KET 50%; AMF + ITZ 66%; AMF + TER 50%. Indifference: 100% AMF + FLU
Santos et al., 2006 [28]	52 *T. rubrum*, 40 *T. mentagrophytes*	CCL + ITZ/KTZ	Ck; 28 °C, 168 h	≥80% inhibition	Synergy: 100%
Sugiura et al., 2021 [29]	8 *T. rubrum*, 8 *T. interdigitale*	EFZ + TER, EFZ + ITZ, LUZ + TER, LUZ + ITZ, TAV + TER, TAV + ITZ, LUZ + TAV	Ck, 35 °C, 96 h	≥80% inhibition	Synergy: EFZ + TER 43.8%, EFZ + ITZ 12.5%, LUZ + ITZ 31.25%, TAV + ITZ 18.7%. Additivism: EFZ + TER 43.75%, EFZ + ITZ 18.75%, LUZ + TER 31.25%, LUZ + ITZ 18.75%, TAV + TER 25%, TAV + ITZ 6.25%. Indifference: EFZ + TER 12.5%, EFZ + ITZ 68.75%, LUZ + TER 68.75%, LUZ + ITZ 50%, TAV + ITZ/TER 75%. LUZ + TAV indifferent effect on some strain
Tamura et al., 2014 [30]	11 *T. rubrum*, 8 *T. Mentagrophytes*, 1 *T. tonsurans*, 1 *T. verrucosum*, 3 *M. gypseum*, 3 *E. floccosum*	AMF + ITZ	Ck; 30 °C, 72–168 h	≥80% inhibition	Synergistic interactions: 25.9% Additivism interactions: 59.2%. Indifference effect: 14.9% No antagonistic effects were detected

GRI, griseofulvin; KTZ, ketoconazole; AMF, amorolfine; TER, terbinafine; FLU, fluconazole; ITZ, itraconazole; MIZ, miconazole; SER, sertaconazole; SUL, sulconazole; CCL, cyclopirox; EFZ, efinaconazole; LUZ, luliconazole; TAV, tavaborole; Ck, Checkerboard titration (performed in broth or otherwise specified). M., *Microsporum*; T., *Trichophyton*; E., *Epidermophyton*. The interaction was defined as synergistic if the FIC index (FICI) was ≤0.5, additivism if >0.50 but <1.0, indifferent if FICI was ≥1 but ≤4.0, and antagonistic if FICI was >4.0.

**Table 2:** Antifungal combinations against dermatophytes: in vitro effects of an antifungal plus a chemical compound other than antifungal.

Reference	Number of Isolates and Species	Combinations	Methods	Reading Endpoint	Results
Danielli et al., 2018 [31]	2 *T. rubrum,* 2 *T. mentagrophytes,* 2 *M. canis,* 2 *M. gypseum*	*Schinus lentiscifolius Marchand +* TER/CCL	Ck, time-kill curves;	100% inhibition	Synergy: EO + TER 50%, EO + CCL 25%. Additivism: EO + TER 37.5%, EO + CCL 62.5%. Indifference: EO + TER 12.5%, EO + CCL 12.5.
Dias et al., 2017 [32]	1 *T. rubrum,* 1 *T. mentagrophytes*	E.O. *L. lusieri*/E.O. *C. citratus* + TER	Fixed ratio combination; 30 °C, 96 h	≥90% inhibition	5% Growth in 1:1 combination EO *L. lusieri* + TER, 20% growth in 1:1 combination EO *C. citratus* + TER
Ala et al., 2010 [33]	1 *T. rubrum*, 1 *T. mentagrophytes*, 1 *T. verrucosum*, 1 *E. flocossum*	Allicin + KTZ/FLU	Ck; 28 °C, 168–240 h	≥50–90% inhibition	Synergy/additivism: 54%, indifference: 46% after 7 days. Synergy/additivism: 33.5%, 66.5%. Indifference: after 10 days.
Galgóczy et al., 2008 [34]	2 *M. canis*, 1 *M. gypseum*, *3 T. mentagrophytes*, 1 *T. rubrum*, 1 *T. tonsurans*	PAF (*Penicillin Chrysogeneum* Antifungal Protein) + FLU	Ck; 37 °C, 96–168–240 h	% of inhibition	Decreased growth when used in combination
Houël et al., 2014 [35]	1 *T. mentagrophytes*, 1 *M. gypseum*	E.O. *Otacanthus azureus* + ITZ/FLU/KTZ	Ck; 32 °C, 120 h	No visible growth	Synergy in *T. mentagrophytes*, indifference in *M. gypseum*
Khan et al., 2011 [36]	1 *T. rubrum*	*S. aromaticum*/eugenol/*C. verum*/cinnamaldehyde/*C. martini*/geraniol + FLU	Ck; 30 °C, 48 h	No visible growth	Synergy: 100% in all combinations
Khan et al., 2014 [37]	1 *T. rubrum*	E.O. *C. copticum* or E.O. *T. vulgaris* or thymol + FLU	Ck; 30 °C, 48 h	No visible growth	Synergy: E.O. *T. vulgaris* or thymol + FLU. Indifference: *C. copticum* + FLU
Khoury et al. 2019, [38]	1 *T. rubrum,* 1 *T. mentagrophytes,* 1 *T. violaceum,* 1 *T. soudanense,* 1 *T. tonsurans*	E.O. *Hitellina lobelii* + FLU/GRI	Ck; 25 °C, 72 h	No visible growth	Synergy in all strains, except for additivity EO + FLU in *T. tonsurans*
Maciel et al., 2019 [39]	3 *T. mentagrophytes*, 2 *T. rubrum*, 1 *M gypseum*	E.O. *Cryptocarya aschersoniana* + TER	Ck; 35 °C, 48 h	No visible growth	Indifference for all strains except for additivism in 1. *T. rubrum*
Pyun et al., 2005 [40]	1 *T. rubrum*, 1 *T. erinacei*, 1 *T. soudanense*	*Allium sativum/Allicin + KTZ*	Ck; 24–28 °C, 72 h	No visible growth	Synergy: *A. sativum* + KTZ 100%. Additivism: *Allicin +* KTZ 100%
Roana et al., 2021 [41]	*1 T. rubrum*	Tea tree oil (TTO) + ITZ/KTZ	Ck; 28–30 °C, 168 h	No visible growth	Synergy with both combinations
Rodriguez et al., 2013 [42]	1 *T. rubrum*	44 extracts from 9 *Baccharis* spp. And 4 flavonoids and 3 ent-clerodanes + TER	HTSS assay, fixed concentration; 28–30 °C, 168 h	No visible growth	Synergy with bacrispine or baccho A + TER
Shin et al., 2004 [43]	1 *T. erinacei*, 1 *T. mentagrophytes*, 1 *T. rubrum*, 1 *T. tonsurans*, 1 *T. schoenleinii*, 1 *T. soudanense*	*P. graveolens* oil, citronellol, and geraniol + KTZ	Ck; 25 °C, 72 h	No visible growth	Synergy: 100%
Shin et al., 2004 [44]	1 *T. erinacei*, 1 *T. mentagrophytes*, 1 *T. rubrum*, 1 *T. schoenleinii*, 1 *T. soudanense*.	E.O. fraction of *A. rugosa* + KTZ	Ck; 25 °C, 72 h	No visible growth	Synergy: 100%
Sim et al., 2008 [45]	1 *T. erinacei*, 1 *T. mentagrophytes*, 1 *T. rubrum*, 1 *T. schoenleinii*, 1 *T. soudanense*, 1 *T. tonsurans*	*Ligustilide/Butylidene phthalide* + ITZ/KTZ	Ck; 25 °C, 72 h	≥50% inhibition	Synergy: 35% Additivism: 65%
Soares et al., 2014 [46]	3 *T. rubrum*, 3 *T. mentagrophytes*	Protocatechuic acids (n = 5) + FLU	Ck; 35 °C, 168 h	≥50% inhibition	Synergy: 1 *T. mentragrophytes* PA9 + FLU. Additivism or indifference in other cases.
Tiwari et al., 2017 [47]	1 *T. mentagrophytes*, 1 *M. canis*	ZnO particles from *Rosa indaca* + KTZ	Disk diffusion; 28 °C, 48 h	Inhibition diameter	Decreased growth when used in combination
Tullio et al., 2019 [48]	1 *T. mentagrophytes*, 1 *M. canis*, 1 *T. rubrum*	E.O. *Menta piperita* + ITZ/KTZ	Ck; 30 °C, 168 h	No visible growth	Synergy in *T. mentagrophytes*, indifference in *M. canis* and *T. rubrum*
Vörös-Horváth et al., 2020 [49]	*1 T. rubrum*	E.O. *Melaleuca* *altifornia* + TIO	Ck; 28 °C, 168 h	No visible growth	Synergy: 100%
Onyewu et al., 2007 [50]	2 *T. mentagrophytes*	cyclosporine A or FK506 + FLU	Ck + ex vivo *T. mentagrophytes human* skin infection model	≥80% inhibition	Synergy in all cases except indifference FKS506+FLU against 1 strain
Ozawa et al., 2005 [51]	5 *T. mentagrophytes*	TAC + ITR	Agar dilution, Ck; 27 °C, 168 h	≥50% inhibition	Synergy: 80%
Zhang et al., 2018 [52]	13 *T. rubrum*, 6 *T. mentagrophytes,* 5 *M. canis*, 4 *E. floccosum*	TAC/TRI + ITZ/TER/BIZ/AMF	Ck; 35 °C, 96–120 h	≥80–100% inhibition	Synergy: TAC/ITZ 39%, TAC/TRB 43%, TAC/BIZ 43%, TRI/ITZ 7%, TRI/BIZ 11%. Indifference in all other cases.
Simonetti et al., 2009 [53]	6 *M. canis*, 6 *T. mentagrophytes*, 10 *T. rubrum,* 2 *M. gypseum*	lipopeptide Pal-Lys-Lys-NH2 (PAL) + FLU/ITZ/TER	Ck; 35 °C, 96 h	≥90% inhibition	Synergy: PAL/TER 52%, PAL/ITZ 67%, PAL/FLU15%. Indifference: PAL/TER 48%, PAL/ITZ 33%, PAL/FLU 85%
Simonetti et al., 2009 [54]	4 *M. canis*, 5 *T. mentagrophytes*, 9 *T. rubrum*, 2 *M. gypseum*	Tachiplesina III + TER	Ck; 35 °C, until visible growth	≥90% inhibition	Synergy: 30% Indifference: 70%
Simonetti et al., 2014 [55]	6 *M. canis*, 6 *T. mentagrophytes,* 8 *T. rubrum*	IB-367 + TER/FLU/ITZ	Ck, time-kill curves; 35 °C, until visible growth	No visible growth	Synergy: *M. canis* IB-367 + FLU 50%, IB-367 + ITZ 17%, IB-367 + TER 33%; *T. mentagrophytes* IB-367 + FLU 33%, IB-367 + ITZ 67%, IB-367 + TER 17%; *T. rubrum* IB-367 + FLU 25%, IB-367 + ITZ 13%, IB-367 + TER 25%
Moriello et al., 2007 [56]	1 *M. canis*	CLO + MIZ	Growth in broth	No visible growth	No growth
Perrins N., et al. 2003 [57]	10 *M. canis*	CLO + MIZ	Agar Dilution: 26 °C, 120 h	No visible growth	Synergy: 50% Additivism: 40% Indifference: 10%
Perrins et al., 2005 [58]	9 *T. mentagrophytes,* 9 *T. erinacei*, 5 *M. persicolor*	CLO + MIZ	Agar dilution; 26 °C, 168 h	No visible growth	Synergy: 8.70% Additivism: 56.52% Indifference: 34.78
Nyilasi et al., 2014 [59]	1 *T. rubrum*, 1 *T. mentagrophytes*, 1 *M. gypseum*, 1 *M. canis*	LOV/SIM/FLV/ROS/ATO/PRA/NYT/PN + AMB/KTZ/ITZ/FLU/TER/GRI	Ck; 30 °C, 96 h	No visible growth	Synergy: 85.92% Indifference: 14.08%
Aneke et al., 2020 [60]	36 *M. canis*	Haloperidol/ promethazine + ITZ/FLU	Ck, disk diffusion, time-kill curve; 30 °C, 48 h	≥80% inhibition	Synergy: ITZ + PRO 91.7%, ITZ + HAL 77.8%, FLU + PRO 25%, FLU + HAL 5.5%. Indifference: ITZ + PRO 8.3%, ITZ + HAL 22.2%, FLU + PRO 47.2%, FLU + HAL 61.2%. Antagonism: FLU + PRO 27.8%, FLU + HAL 33.1%.

TER, terbinafine; CCL, ciclopirox; KTZ, ketoconazole; FLU, fluconazole; ITZ, itraconazole; GRI, griseofulvin; TAC, tacrolimus; TRI, triamcinolone acetonide, BIZ, bifonazole; AMF, amorolfine; CLO, chlorhexidine; MIZ, miconazole; LOZ, lovastatin; SIM, simvastatin; FLV, fluvastatin; ROS, rosuvastatin; ATO, atorvastatin; PRA, pravastatin; NYT, nystatin; PN, prymicin. Ck, Checkerboard titration (performed in broth or otherwise specified). M., *Microsporum*; T., *Trichophyton*; E., *Epidermophyton*. E.O., essential oil. The interaction was defined as synergistic if the FIC index (FICI) was ≤0.5, additivism if >0.50 but <1.0, indifferent if FICI was ≥1 but ≤4.0, and antagonistic if FICI was >4.0.

**Table 3:** Antifungal combinations against dermatophytes: clinical cases.

Reference	Number of Isolates and Species	Combinations	Results
Adamski et al., 2014 [61]	A 34-year-old Polish Caucasian male with erythematous, exfoliating, clearly distinct lesion located on the index finger of the right hand caused by *T. rubrum*	ITZ daily dose 100 mg and topical IMZ at first; subsequently the topical drug was switched to a pyridinone derivative	Full recovery
Budihardja et al., 2010 [62]	45-year-old patient, renal transplant recipient with widespread erosive tinea corporis caused by *T. mentagrophytes*	TER daily plus CCL olamine topically for 9 weeks	Clinical cure
Czaika et al., 2013 [63]	Two girls (11 and 7 years) with zoophile tinea faciei and tinea corporis due to *T. mentagrophytes*	Systemic TER at a daily dose of 125 mg, based on body weight for 5 weeks (11-year-old girl) and for 4 weeks (7-year-old girl) was prescribed. Twice daily, application of ISZ/DFV cream containing ISN 1% and DFV 0.1% was prescribed for 10 days (facial lesion) or 14 days (other lesions), subsequently to be continued with CCL.	Improvement of all lesions and pruritus in both patients 2 weeks after treatment initiation
Durant et al., 2009 [64]	A 31-year-old patient presented with a diagnosis of granulomatous dermatophytosis due to *T. rubrum*	ITZ plus TER 250 mg	No improvement
Fabrizi et al., 2017 [65]	A 74-years-old with interdigital tinea pedis and distal-lateral onychomycosis of both big toes were present due to *T. rubrum* and *Tyrophagus putrescentiae*	TER 250 mg/day and CCL 8% nail lacquer for 16 weeks	Full recovery
Ghislanzoni, 2008 [66]	A 35-year-old male with tinea incognito due to *T. rubrum*	Topic ISZ plus DFC for 4 weeks	Partial improvement
Hsieh et al., 2019 [67]	A 60-year-old man and a 51-year-old-woman with disseminated tinea corporis caused by *T. mentagrophytes*	ITZ with topical EBE	Full Recovery
Jang et al., 2017 [68]	A 9-year-old male with kerion celsi caused by *T. erinacei*	TER 250 mg/day for 6 weeks and MTP 12 mg/day for the first week.	Full recovery
Khaled et al., 2007 [69]	A 6-year-old Tunisian boy with tinea favosa due to *T. schoenleinii*	20 mg/kg/day of oral GRI 400 mg twice daily for 6 weeks and topical IMZ for 8 weeks	Full recovery
Kimura et al., 2020 [70]	A 27-year-old Nepalese woman with extensive dermatophytosis caused by *T. mentagrophytes*/*T. interdigitale*	Oral ITZ 100 mg/day and topical LUZ	Full recovery
Kotrekhova, 2008 [71]	A 61-year-old male with inguino-femoral skin fold mycosis due to *T. rubrum*	Topic ISZ plus DFC for 4 weeks	Clinical improvement and eradication
Lacaz et al., 1999 [72]	One patient with dermatophytosis caused by *T. raubitschekii*	FLU 150 mg per os/week for 4 weeks plus topical ISZ	Recurrence of lesions after the medication was discontinued.
Lee et al., 2008 [73]	A 68-year-old male teacher with tinea corporis due to *T. rubrum*	Two treatments: topical cream containing a combination of CTZ 10 mg and HDC for 3 weeks; topical cream ISZ plus DFV for 2 weeks.	Recurrence of skin infection after the first treatment; improvement with cream ISZ/DFV
Lin et al., 2014 [74]	A 58-year-old male with *Trichophyton* spp. Endoftalmitis	Intravitreal AMP B 5 μg/0.1 mL injection and oral VOR 200 mg twice daily + surgery	Visual acuity improvement
Papini et al., 2004 [75]	A 22-year-old black male student with onychomycosis due to *T. raubitschekii*	Oral TER 250 mg/day and CYC nail lacquer for 8 weeks.	Full recovery
Pietrzak et al., 2012 [76]	A woman with dermatophytosis of the thighs due to *T. mentagrophytes*	ISZ and DFV; cryotherapy with liquid nitrogen was started after antifungal therapy, for persistent lesions of the skin	Direct microscopic mycologic examination and culture on BioMerieux medium were negative; however, the lesions persisted, assuming a completely different aspect. recovery after cryotherapy.
Markey et al., 2003 [77]	Two young sisters, ages 5 and 6 years with tinea capitis due to *T. soudanense*	GRI 15 mg/kg/day and 2.5% SES lotion as a shampoo twice a week for 8 weeks for the tinea capitis	Full recovery
Calabrò et al., 2011 [78]	A 26-year-old man born in Senegal, but living in Naples for seven months with *T. violaceum* infection	Systemic treatment with GRI at 15 mg/kg/day and topical with TIO 1% Twice a day for one month were administered.	Full recovery
Balci et al., 2008 [79]	A 54-year-old immunocompetent female with widespread, chronic, and fluconazole-resistant *T. rubrum* Infection	Systemic ITZ and SRZ cream	Full Recovery
Veraldi et al., 2015 [80]	A 47-year-old Italian woman with tinea imbricata located on the thighs and legs due to *T. concentricum*	GRI 1 g/day for 6 weeks and 1% TER cream 2 applications/day for 6 weeks	Full recovery
Yin, et al., 2013 [81]	Three familial cases with tinea capitis and tinea corporis due to *M. canis*	Oral TER + cream containing 1% NAF 025% KTZ-100 mg/day ITZ + cream containing 1% NAF 025% KTZ	Full recovery
Zhan et al., 2015 [82]	A 48-year-old female with a chronic disseminated dermatophytosis due to *T. violaceum*	TER 0.25 g/day, 1% TER gel for external use and 2% KTZ lotion for shampoo and bath	A sufficient decrease of the scalp and skin damage after 4 weeks, but no improvement of the nails, and after that, the patients was lost to follow-up.
Zhang et al., 2009 [83]	Three family members with kerion and tinea corporis due to *T. mentagrophytes*	ITZ 100 mg/day plus KTZ shampoo 2% + 3 months	Clinical cure
Zhang et al., 2015 [84]	A 54-year-old Chinese male patient with generalized superficial mycosis caused by *T. raubitschekii*	TER 250mg/day and topical NHY and KTZ cream, containing 1% NHY and 0.25% KTZ.	Full recovery
Zhuang et al., 2016 [85]	An 18-year-old girl with tinea faciei on the right eyebrow caused by *T. mentagrophytes*	TER 250 mg/day combined with daily topical use of 1% naftifine–0.25% ketoconazole cream, after washing the lesion with 2% ketoconazole shampoo.	Full recovery
Abdulkarim et al., 2006 [86]	Five cases report of fungal keratitis caused by *T. schoenleinii*	Case 2: hourly topical NAT 50 mg/mL and OFL 3 mg/mL 4 times daily and oral FLU 200 mg twice daily. Case 3: topical AMP B 10 mg/mL every 30 min for 1 day and hourly thereafter, MIZ 10 mg/mL hourly, and OFL 3 mg/mL 4 times daily, along with oral FLU 200 mg twice daily. Case 4: hourly topical MIZ 10 mg/mL, oral FLU 200 mg twice daily for 3 days and once daily thereafter. Because of a worsening clinical course, topical AMP B 5 mg/mL was added hourly. Case 5: hourly topical NAT 50 mg/mL and oral FLU 200 mg twice daily. Following gradual improvement in the stromal infiltrate, cessation of further stromal thinning, and resolution of the hypopyon.	Improvement
Romano et al., 2014 [87]	18 children and 18 adults with infections due to *T. violaceum*	The 13 index cases and the 16 patients infected by them were treated with 10∫mg/kg day GRI for 45 days and topical IMZ for 20–30 days. 23 adults with spreading tinea corporis were treated with 100 mg ITZ for 15–20 days and those with tinea capitis with the same dose of the antimycotic for 45 days and with topical IMZ for 15–20 days, depending on the number of patches.	Full recovery
Erbakan et al., 1974 [88]	A total of 254 patients with tinea inguinalis, corporis, pedis, manus: 69 *T*. *rubrum*, 31 *T. mentagrophytes*, 7 *T. violaceum*, 18 *E. floccousm*; 6 *M. canis*; no growth in the remaining cases	Topical (i.e., Wilkinson’s salve, iodize alcohol, undecylenic acid, 5-bromo-4′-chlorosalicylanilide, tolnaftate) plus GRI topical vs GRI alone	Topical treatment plus GRI possibly enhances the healing capacity and shortens the time of treatment but no effect in the recurrences
Baran et al., 2007 [89]	Clinical trial AMF plus TER vs. TER alone in 249 patients with onychomycosis with matrix involvement due to *T. rubrum* > 90% of cases	AMF nail lacquer once weekly for 12 months plus TER 250 mg once daily for 3 months	Higher success rate for patients in combination therapy: 59.2% vs 45%
Hussain et al., 1999 [90]	Clinical trial PRE plus GRI in 30 patients with *Trichophyton* infection	Oral GRI and oral PRE	No difference
Baran, 2001 [91]	Clinical trial AMF plus ITZ vs ITZ in 131 patients with *T. rubrum* in the majority of cases	15 months of once-weekly topical AMF lacquer in combination with 6 weeks (group at 6) or 12 weeks (group at 12) of oral TER 250 mg once daily	AMF plus TER is more effective than TER alone

ITZ, itraconazole; LUZ, luliconazole; EBE, eberconazole; TER, terbinafine; CCL, ciclopirox; MTP, methylprednisolone; NHY, naftifine hydrochloride; GRI, griseofulvin; AMP B, amphotericin B; AMR, amorolfine; VOR, voriconazole; ISN, isoconazole nitrate; DFV, diflucortolone valerate; ISZ, isoconazole; DFC, difluocortolone; CTZ, clotrimazole; HDC, hydrocortisone; NAF, naftifine; CYC, ciclopirox olamine; IMZ, imidazole; SRZ, sertaconazole nitrate; TIO, tioconazole; SES, selenium sulphide; NAT, natamycin; OFL, ofloxacin; PRE, prednisolone. M., *Microsporum*; T., *Trichophyton*; E., *Epidermophyton*.
